# Protective Effects of Mixed Probiotics, Oralis SB^®^, on the Ligation-Induced Experimental Periodontitis and Alveolar Bone Loss in Rats

**DOI:** 10.3390/antiox15070804

**Published:** 2026-06-27

**Authors:** Su Yeon Kim, Eun Hye Han, Hyun Su Park, Jae-Kwang Kim, Sae-Kwang Ku

**Affiliations:** 1 Department of Anatomy and Histology, College of Korean Medicine, Daegu Haany University, Gyeongsan 38610, Republic of Korea; ksuy.1126@dhu.ac.kr; 2 Food Ingredients Research Team, R&D Center, Dong-A Pharmaceutical, Yongin 17073, Republic of Korea; ehhan@donga.co.kr; 3 DENTIS Co., Ltd., Daegu 41065, Republic of Korea; hyunsu@dentis.co.kr; 4 Department of Biomaterials Science, College of Natural Resources & Life Science, Pusan National University, Miryang 50463, Republic of Korea; 5 Department of Physiology, College of Korean Medicine, Daegu Haany University, Gyeongsan 38610, Republic of Korea

**Keywords:** periodontitis, Oralis SB^®^, inflammation, alveolar bone loss, epithelial barrier dysfunction

## Abstract

The present study evaluated the protective effects of Oralis SB^®^ (Osb), a commercial multi-strain probiotic formulation, in a ligature-induced experimental periodontitis (EPD) Sprague–Dawley rat model. Male rats were subjected to ligature placement and orally administered Osb (1.25 × 10^8^, 1.25 × 10^9^, and 6.25 × 10^9^ CFU/head) or received single-strain treatments with each of the four constituent strains (1.25 × 10^9^ CFU/head) for 10 days. Periodontal changes were assessed by microbiological, biochemical, histological, and bone-related analyses. Ligature placement markedly increased anaerobic bacterial counts, neutrophil-associated myeloperoxidase activity, and inflammatory mediator production in gingival tissues, accompanied by elevated gingival IgA levels and impaired expression of epithelial tight junction-related genes, including claudin-1, claudin-5, occludin, and zonula occludens-1. Osb administration significantly attenuated bacterial overgrowth, restored gingival IgA contents, and suppressed the production of inflammatory mediators. Osb also reduced malondialdehyde level and inducible nitric oxide synthase activity, as markers of oxidative and nitrosative stress, and decreased metalloproteinase-8 expression in gingival tissue. In addition, Osb effectively prevented alveolar bone deterioration by improving focal bone mineral density, reducing osteoclast numbers, and restoring the receptor activator of nuclear factor-κB/osteoprotegerin balance. Histological evaluation further confirmed that Osb alleviated gingival inflammation and alveolar bone loss. Notably, the lowest dose of Osb (1.25 × 10^8^ CFU/head) consistently produced significantly greater protective effects than each of the four individual component strains across all measured parameters. Osb exerted protective effects against EPD by modulating anaerobic microbial overgrowth, inflammatory and oxidative responses, epithelial barrier integrity, and alveolar bone resorption. These findings support the therapeutic potential of Osb in periodontitis.

## 1. Introduction

Periodontitis is a chronic inflammatory disease that causes progressive destruction of the tooth-supporting tissues, making it a major cause of tooth loss worldwide. The disease is initiated by a dysbiotic shift in the oral microbiota, characterized by an overgrowth of pathogenic anaerobes such as *Porphyromonas gingivalis*, *Tannerella forsythia*, and *Treponema denticola* [1]. This imbalance elicits an exaggerated host immune response, characterized by excessive recruitment and activation of neutrophils, which release reactive oxygen species, proteolytic enzymes, and pro-inflammatory mediators that drive connective tissue breakdown and alveolar bone resorption [2]. Beyond its local effects, periodontitis has been increasingly linked to systemic conditions such as cardiovascular disease, highlighting its broader public health and socioeconomic impact [3,4].

The gingival epithelium serves as the primary physical barrier that restricts bacterial invasion, and consequently suppresses the inflammatory response, thereby maintaining periodontal homeostasis [5]. However, changes in the oral microbiota toward pathogenic anaerobic bacteria in periodontitis profoundly disrupt this barrier by altering epithelial cell function and impairing key tight junction components [6]. *P. gingivalis*, a keystone periodontal bacterium, produces virulence factors such as lipopolysaccharide (LPS) and gingipains, which have been shown to disrupt epithelial barrier integrity by altering the expression of tight junction components, including claudins, occludin, and ZO-1 [7,8]. Increased epithelial permeability allows microbial products to reach the underlying connective tissues, amplifying neutrophil recruitment and cytokine release and accelerating periodontal tissue destruction. Thus, disruption of epithelial tight junctions is considered an early, pivotal event that links microbial imbalance to subsequent periodontal breakdown.

While mechanical debridement using scaling and root planing (SRP) is the gold standard for periodontal therapy, its effectiveness is often limited by restricted instrument access to deep pockets and complex root anatomy, which can promote the rapid recolonization of bacteria [9]. Furthermore, the adjunctive use of antibiotics is associated with notable limitations, including adverse effects, antimicrobial resistance, and disruption of the normal microbiota, prompting increasing interest in alternative therapeutic strategies [10,11]. In this context, probiotics have attracted interest as a potential adjunctive approach for periodontal management. Probiotics may exert beneficial effects on periodontal health by shifting the oral microbiota from a pathogenic to a more health-associated composition, while simultaneously regulating inflammatory responses and host immune function [12]. Consistent with these findings, meta-analyses confirmed that adjuvant probiotic therapy significantly improves probing pocket depth and clinical attachment loss compared with SRP alone in patients with periodontitis, without serious adverse effects [13,14].

Oralis SB^®^ (Osb) is a commercialized multi-strain probiotic formulation composed of *Lactobacillus helveticus* Rosell-52 (Lh), *Lacticaseibacillus rhamnosus* Rosell-11 (Lr), *Saccharomyces cerevisiae* var. *boulardii* CNCM I-1079 (Sb), and *Bifidobacterium longum* Rosell-175 (Bl). To date, Osb has been evaluated in 13 clinical studies related to oral health, including two studies in periodontitis [15]. In these periodontal studies, probiotic mouthwash using Osb improved plaque and gingival index, periodontal pocket depth, and clinical attachment [16,17]. Although several clinical studies have demonstrated the safety and benefits of Osb, animal studies are still required to elucidate its disease-specific mechanisms in periodontitis that cannot be fully addressed in humans. Therefore, the present study investigated the protective effects of Osb in a ligature-induced experimental periodontitis (EPD) rat model, with a focus on periodontal inflammation, alveolar bone loss, and barrier dysfunction. In addition, we examined whether a combined multi-strain formulation (Osb) provides greater efficacy than the individual strains administered separately.

## 2. Materials and Methods

### 2.1. Preparation of Test Materials

Powders of four probiotic strains (Lh, Lr, Sb, and Bl) as well as a commercial probiotic formulation (Osb) containing these four strains, were manufactured by Lallemand Health Solutions Inc. (Mirabel, Quebec, Canada) and supplied via DENTIS (Daegu, Republic of Korea). Lh, Lr, Sb, and Bl powders were suspended in distilled water at a concentration of 16.05 mg/mL. Osb was suspended in distilled water at concentrations of 1.605, 16.05, and 80.15 mg/mL. Indomethacin (IND; Sigma-Aldrich, St. Louis, MO, USA) was dissolved in distilled water at a concentration of 1 mg/mL. All test materials were stored in a refrigerator at 4 °C until use.

### 2.2. Animal Husbandry

A total of one hundred male Sprague–Dawley (SD) rats (6 weeks old) were supplied by OrientBio (Seongnam, Republic of Korea). The animals were housed in polycarbonate cages at a density of five rats per cage under controlled environmental conditions (temperature 20–25 °C, humidity 50–55%) with a 12 h light/dark cycle. All rats were provided with standard chow and had free access to water *ad libitum*. After ten days of acclimatization, rats were divided into 10 groups (10 rats per group): Intact vehicle control, EPD control, IND (IND 5 mg/kg), Lh-M (Lh single-formula 1.25 × 10^9^ CFU/head), Lr-M (Lr single-formula 1.25 × 10^9^ CFU/head), Sb-M (Sb single-formula 1.25 × 10^9^ CFU/head), Bl-M (Bl single-formula 1.25 × 10^9^ CFU/head), Osb-L (1.25 × 10^8^ CFU/head), Osb-M (1.25 × 10^9^ CFU/head), and Osb-H (6.25 × 10^9^ CFU/head). Body weight was recorded daily from one day before ligature placement until the end of the 10-day administration period. All animal experimental procedures were conducted in accordance with national guidelines for the care and use of laboratory animals and were approved by the Institutional Animal Care and Use Committee of Daegu Haany University (Approval No. DHU-2025-015, Gyeongsan, Republic of Korea).

### 2.3. Induction of EPD

EPD was induced by placing a nylon thread ligature (AILEE, Busan, Republic of Korea) around the cervix of the upper left incisor under inhalation anesthesia with 2–3% isoflurane (Hana Pharm. Co., Hwaseong, Republic of Korea) as previously described [18,19]. The ligature was secured on the buccal side of the tooth, resulting in a subgingival position on the palatal side and a supragingival position on the buccal side. In the intact vehicle control group, ligation was not performed; instead, only the cervix of the upper left incisor was identified during the experimental procedures.

### 2.4. Measurements of Alveolar Bone Loss

After euthanasia, the maxilla containing the ligature site was excised following photographic captures. Horizontal alveolar bone loss–defined as the distance between the cusp tip and the alveolar bone crest–was measured along the axis of the root of the upper left incisor using an electronic digital caliper as previously reported [18,19].

### 2.5. Measurement of Bone Mineral Density (BMD)

Mean focal BMDs in the maxillary incisor root regions were measured using a DEXA scanner (InAlyzer, Medikors, Seongnam, Republic of Korea) and expressed as g/cm^2^ for all groups.

### 2.6. Microbiological Analysis

Buccal gingival tissues adjacent to the ligated upper left incisor were excised and transferred into 0.3 mL of brain heart infusion (BHI) broth. The tissues were immediately homogenized using mechanical disruption and sonication. Homogenates were serially diluted and spread onto BHI agar supplemented with sodium propionate, lithium chloride, cysteine hydrochloride, and defibrinated sheep blood. The plates were incubated under anaerobic conditions at 37 °C for 48 h, and CFUs were then enumerated and expressed as ×10^2^ CFU/g tissue.

### 2.7. Quantification of Myeloperoxidase (MPO) Activity

Gingival tissues around the ligature site were homogenized in 0.5% hexadecyltrimethylammonium bromide in 50 mM phosphate buffer (pH 6.0), followed by a freeze–thaw cycle and centrifugation. An aliquot of the supernatant was mixed with phosphate buffer containing *o*-dianisidine and hydrogen peroxide, and the absorbance at 460 nm was measured. MPO activity was quantified from the rate of hydrogen peroxide degradation, where one enzymatic unit was defined as the amount of enzyme required to catalyze the conversion of 1 μM H_2_O_2_ per minute at 25 °C. Final values were normalized to tissue weight and expressed as U/mg tissue.

### 2.8. Enzyme-Linked Immunosorbent Assay (ELISA)

Levels of IgA (MBS705069, MyBioSource, San Diego, CA, USA), prostaglandin E_2_ (PGE_2_; KGE004, R&D Systems, Minneapolis, MN, USA), Matrix Metalloproteinase-8 (MMP-8; MBS721664, MyBioSource), tumor necrosis factor-α (TNF-α; ab46070, Abcam, Cambridge, UK), and interleukin-1β (IL-1β; ab100768, Abcam) in buccal gingival tissue homogenates were quantified using commercial ELISA kits, following manufacturers’ instructions. Results were expressed as pg/mg tissue for PGE_2_, TNF-α, and IL-1β, and as ng/mg tissue for IgA and MMP-8.

### 2.9. Measurement of Malondialdehyde (MDA) Content

MDA levels in buccal gingival tissues around the ligature site were measured as an index of lipid peroxidation using a thiobarbituric acid-reactive substances (TBARS) assay. Briefly, tissues were homogenized in 50 mM Tris-HCl buffer (pH 7.4) containing EGTA and phenylmethylsulfonyl fluoride. Aliquots of the homogenate were mixed with a reaction solution composed of sodium dodecyl sulfate, acetic acid, and thiobarbituric acid, heated at 95 °C for 1 h, and then centrifuged. The absorbance of the resulting supernatant was read at 650 nm with a UV/VIS spectrophotometer, and MDA levels were expressed relative to tissue weight (µM/mg).

### 2.10. Measurement of Inducible Nitric Oxide Synthase (iNOS) Activity

Gingival iNOS activity was evaluated by a radiometric L-arginine to L-citrulline conversion assay, adapted from a previous report [20]. Gingival tissues were homogenized, and aliquots were incubated at 22 °C for 30 min in a reaction mixture containing L-[^3^H]-arginine (10 mM, 5 kBq/tube), NADPH (1 mM), calmodulin (30 nM), tetrahydrobiopterin (5 mM), and Ca^2+^ (2 mM). To distinguish specific enzymatic activity, parallel incubations were performed either without NADPH, to estimate non-enzymatic background, or in Ca^2+^-free conditions with excess EGTA, to assess calcium-independent NOS activity. Reactions were terminated by adding ice-cold HEPES buffer (pH 5.5) containing EGTA and EDTA. Samples were passed through Dowex 50W ion-exchange columns. The radioactivity of eluted L-[^3^H]-citrulline was measured by liquid scintillation counting. iNOS activity was expressed as fM of L-[^3^H]-citrulline formed per mg tissue per min.

### 2.11. Quantitative Real-Time Polymerase Chain Reaction (qPCR)

mRNA expression in maxillary gingival tissues was quantified by real-time PCR, following previously described procedures [18,19]. Total RNA was isolated using TRIzol reagent (Invitrogen, Carlsbad, CA, USA) and treated with DNase I (DNA-free DNA Removal Kit, Thermo Fisher Scientific, Rockford, IL, USA) to remove genomic DNA contamination. cDNA was synthesized from purified RNA using the High-Capacity cDNA Reverse Transcription Kit (Thermo Fisher Scientific) according to the manufacturer’s instructions. Quantitative PCR was performed on a CFX96^TM^ Real-Time System (Bio-Rad, Hercules, CA, USA). mRNA expression of each gene was normalized to β-actin mRNA, and relative expression levels were calculated using the comparative threshold cycle method [21]. Primer sequences are listed in Table 1.

### 2.12. Histopathological Analysis

The maxillary segment containing the left and right upper incisors and the ligature site was excised and fixed in 10% neutral-buffered formalin. After fixation, specimens were decalcified in a formic acid and sodium hydroxide solution that was renewed daily for 5 days, then trimmed crossly to include both incisors, embedded in paraffin, and sectioned at 3–4 µm thickness using a microtome. Sections were stained with hematoxylin and eosin, according to previously established methods [18,19].

Histological evaluation focused on the region between the left and right upper incisors corresponding to the ligature placement site. Gingival and alveolar areas were examined by a histopathologist blinded to group allocation. Periodontal damage in these regions was scored on a 0–3 scale based on inflammatory cell infiltration and the integrity of cementum and alveolar bone, as described previously [18,19,22]. Histomorphometric measurements were additionally performed to determine inflammatory cell infiltration (cells/mm^2^) in gingival tissues adjacent to the left incisor, as well as alveolar bone volume (%/mm^2^), osteoclast number (cells/mm^2^), and osteoclast-occupied bone surface (OC/BS, %) in the alveolar bone region between the upper incisors.

### 2.13. Statistical Analysis

All data are expressed as mean ± standard deviation (SD) of ten rats. Group differences were analyzed using one-way analysis of variance (ANOVA) when the assumption of homogeneity of variance (Levene test) was satisfied, followed by Tukey’s honestly significant difference (HSD) post hoc test. When this assumption was violated, Dunnett’s T3 test was conducted. A *p*-value < 0.05 was considered statistically significant. Statistical analyses were performed using SPSS (version 18.0; SPSS Inc., Chicago, IL, USA).

## 3. Results

### 3.1. Experimental Timeline and Body Weight Monitoring

**Figure 1:**
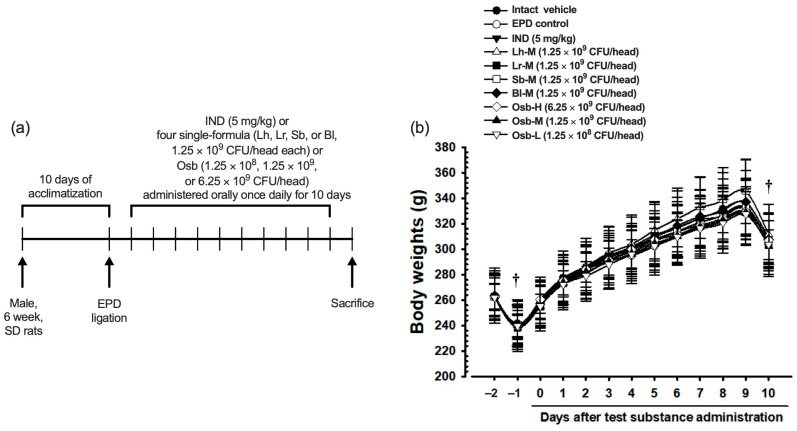
Experimental timeline and body weight changes. (**a**) After ligature placement, rats received daily oral administration of IND (5 mg/kg), four single-formula (Lh, Lr, Sb, or Bl 1.25 × 10^9^ CFU/head), or Osb (1.25 × 10^8^, 1.25 × 10^9^, and 6.25 × 10^9^ CFU/head) for 10 days, while control groups received distilled water. (**b**) Body weight was recorded once daily throughout the experiment. ^†^ All animals were fasted overnight before ligature placement and sacrifice. EPD, experimental periodontitis; IND, Indomethacin; Lh, *Lactobacillus helveticus* Rosell-52; Lr, *Lacticaseibacillus rhamnosus* Rosell-11; Sb, *Saccharomyces cerevisiae* var. *boulardii* CNCM I-1079; Bl, *Bifidobacterium longum* Rosell-175; Osb, Oralis SB^®^.

24 h after the ligature placement, rats received their designated treatments once daily for 10 consecutive days. The treatment groups included distilled water (intact and EPD controls, 2 mL/head), IND (5 mg/kg), single-formula preparations including Lh, Lr, Sb, and Bl (1.25 × 10^9^ CFU/head), and Osb (1.25 × 10^8^, 1.25 × 10^9^, and 6.25 × 10^9^ CFU/head). All animals were euthanized 24 h after the final administration (Figure 1a).

Body weights were monitored daily, beginning one day prior to ligature placement and continuing throughout the 11 days of the whole experimental period. The EPD control group exhibited no significant changes in body weights compared with the intact vehicle control group. Likewise, administration of Osb at any dose, IND, or the single-formula preparations did not result in significant changes in body weights compared with the EPD control group (Figure 1b).

### 3.2. Osb Reduced Bacterial Overgrowth and Modulated Gingival IgA and MPO Activity in EPD Rats

**Figure 2:**
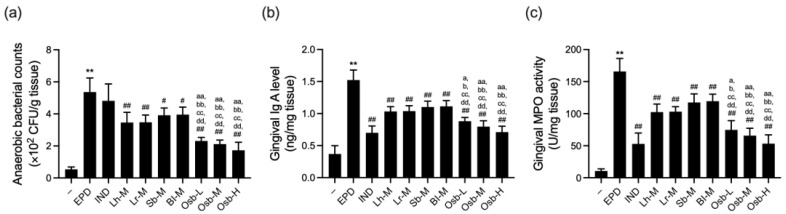
Effects of Osb on anaerobic bacterial counts, gingival IgA levels, and myeloperoxidase (MPO) activity in EPD rats. (**a**) Anaerobic bacterial counts. After anaerobic culture of homogenized buccal gingival tissue, colony-forming units (CFUs) were measured. (**b**) Gingival IgA content. IgA concentrations in gingival tissue homogenates were determined using a commercial enzyme-linked immunosorbent assay (ELISA) kit. (**c**) MPO activity was measured using a colorimetric assay that quantifies the rate of hydrogen peroxide degradation. Data are presented as mean ± SD (*n* = 10/group). Significant vs. intact vehicle control, ** *p* < 0.01; vs. EPD control, ^#^ *p* < 0.05, ^##^ *p* < 0.01; vs. Lh-M, ^a^ *p* < 0.05, ^aa^ *p* < 0.01; vs. Lr-M, ^b^ *p* < 0.05, ^bb^ *p* < 0.01; vs. Sb-M, ^cc^ *p* < 0.01; vs. Bl-M, ^dd^ *p* < 0.01.

Anaerobic bacterial counts, determined from homogenized buccal gingival tissues collected around the ligature site, were markedly elevated in the EPD control group compared with the intact vehicle control group. Oral administration of Osb at all tested doses (1.25 × 10^8^, 1.25 × 10^9^, and 6.25 × 10^9^ CFU/head) significantly reduced these bacterial levels, with the 1.25 × 10^8^ CFU/head dose (Osb-L) showing a greater reduction relative to the single-formula treatments of Lh, Lr, Sb, or Bl (Figure 2a). Consistent with the reduction in bacterial burden, gingival IgA levels, as an indicator of local mucosal immune activation, were also markedly increased in the EPD control group but significantly decreased by Osb treatment at all doses, again with Osb-L demonstrating superior efficacy compared with the single-formula groups (Figure 2b). MPO activity, assessed as a marker of neutrophil accumulation, was markedly elevated in the EPD control group compared with the intact vehicle group. Osb administration significantly reduced MPO activity at all tested doses. Notably, the 1.25 × 10^8^ CFU/head (Osb-L) exerted a stronger protective effect than the single-formula treatments of Lh, Lr, Sb, or Bl (Figure 2c).

### 3.3. Osb Suppressed the Production of Pro-Inflammatory Mediators in Gingival Tissues of EPD Rats

**Figure 3:**
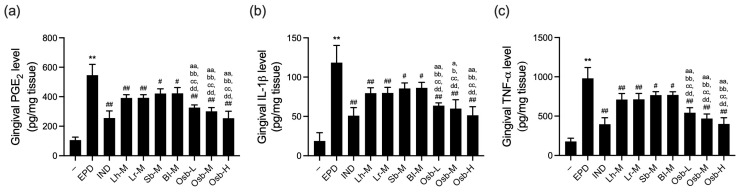
Effects of Osb on gingival pro-inflammatory mediators in EPD rats. (**a**) Prostaglandin E_2_ (PGE_2_), (**b**) interleukin-1β (IL-1β), and (**c**) tumor necrosis factor-α (TNF-α) were measured in gingival tissue homogenates using commercial ELISA kits. Data are presented as mean ± SD (*n* = 10/group). Significant vs. intact vehicle control, ** *p* < 0.01; vs. EPD control, ^#^ *p* < 0.05, ^##^ *p* < 0.01; vs. Lh-M, ^a^ *p* < 0.05, ^aa^ *p* < 0.01; vs. Lr-M, ^b^ *p* < 0.05, ^bb^ *p* < 0.01; vs. Sb-M, ^cc^ *p* < 0.01; vs. Bl-M, ^dd^ *p* < 0.01.

Pro-inflammatory mediators, including PGE_2_, IL-1β, and TNF-α, were significantly elevated in the gingival tissues of the EPD control group compared with the intact vehicle control group (Figure 3). Oral administration of Osb at all tested doses (1.25 × 10^8^, 1.25 × 10^9^, and 6.25 × 10^9^ CFU/head) significantly reduced these pro-inflammatory mediator levels. Notably, the Osb-L (1.25 × 10^8^ CFU/head) showed significantly greater suppressive effects than the single-formula treatment of Lh, Lr, Sb, or Bl.

### 3.4. Osb Reduced Gingival MMP-8 Expression in EPD Rats

**Figure 4:**
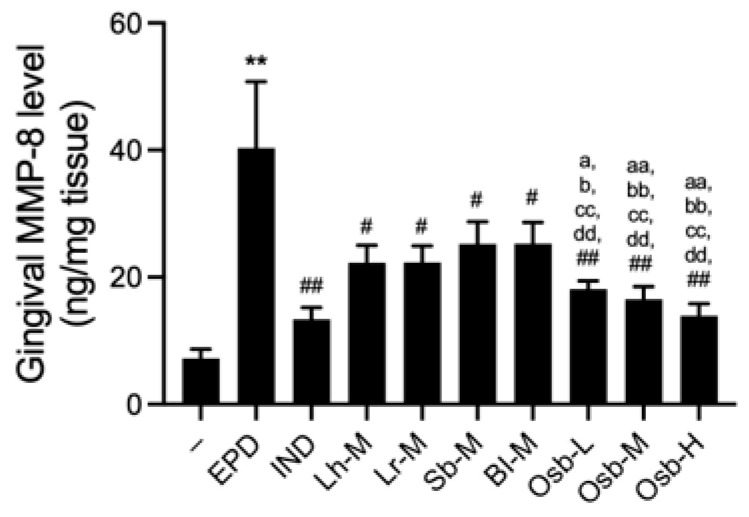
Effects of Osb on gingival matrix metalloproteinase-8 (MMP-8) in EPD rats. Gingival MMP-8 concentrations were measured in tissue homogenates using a commercial ELISA kit. Data are presented as mean ± SD (*n* = 10/group). Significant vs. intact vehicle control, ** *p* < 0.01; vs. EPD control, ^#^ *p* < 0.05, ^##^ *p* < 0.01; vs. Lh-M, ^a^ *p* < 0.05, ^aa^ *p* < 0.01; vs. Lr-M, ^b^ *p* < 0.05, ^bb^ *p* < 0.01; vs. Sb-M, ^cc^ *p* < 0.01; vs. Bl-M, ^dd^ *p* < 0.01.

MMP-8, a neutrophil-derived collagenase involved in extracellular matrix degradation, was significantly elevated in the gingival tissues of the EPD control group compared with the intact vehicle control group (Figure 4). Oral administration of Osb at all doses (1.25 × 10^8^, 1.25 × 10^9^, and 6.25 × 10^9^ CFU/head) significantly reduced MMP-8 expression. Notably, the Osb-L (1.25 × 10^8^ CFU/head) showed a significantly greater reduction in MMP-8 levels than the single-formula treatments of Lh, Lr, Sb, or Bl.

### 3.5. Osb Restored Tight Junction-Related mRNA Expression in EPD Rats

**Figure 5:**
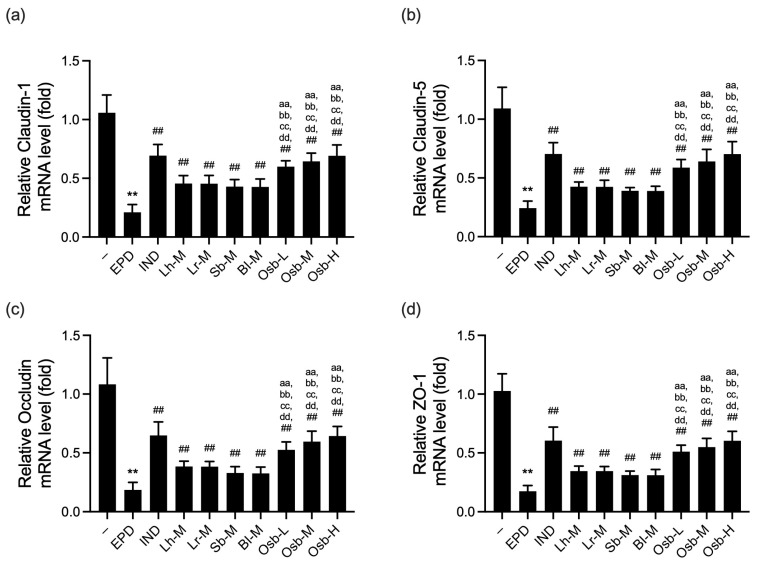
Effects of Osb on gingival tight junction-related gene expressions in EPD rats. Relative mRNA levels of (**a**) claudin-1, (**b**) claudin-5, (**c**) occludin, and (**d**) zonula occludens (ZO-1) in gingival tissue were quantified by reverse transcription-quantitative PCR (RT-qPCR) and normalized to β-actin mRNA expression. Data are presented as mean ± SD (*n* = 10/group). Significant vs. intact vehicle control, ** *p* < 0.01; vs. EPD control, ^##^ *p* < 0.01; vs. Lh-M, ^aa^ *p* < 0.01; vs. Lr-M, ^bb^ *p* < 0.01; vs. Sb-M, ^cc^ *p* < 0.01; vs. Bl-M, ^dd^ *p* < 0.01.

Gingival tight junction integrity was significantly disrupted in EPD control rats, as reflected by a significant decrease in the mRNA expression of claudin-1, claudin-5, occludin, and ZO-1 compared with intact vehicle controls (Figure 5). Oral administration of Osb at all tested doses (1.25 × 10^8^, 1.25 × 10^9^, and 6.25 × 10^9^ CFU/head) significantly increased the mRNA expression of these tight junction-related genes. Notably, the lowest dose (Osb-L, 1.25 × 10^8^ CFU/head) restored mRNA expression levels greater than those observed with single-formula treatment (Lh, Lr, Sb, or Bl), suggesting a superior barrier-protecting effect of the Osb combination.

### 3.6. Osb Reduced Oxidative and Nitrosative Stress in Gingival Tissue

**Figure 6:**
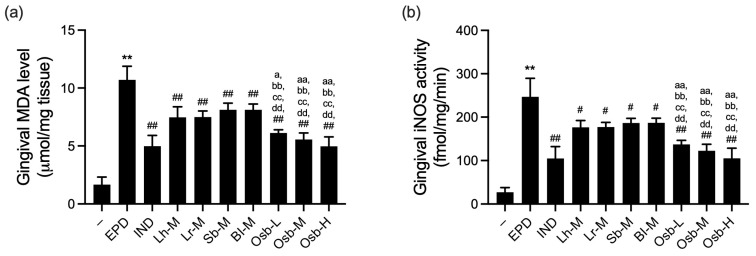
Effects of Osb on gingival malondialdehyde (MDA) levels and inducible nitric oxide synthase (iNOS) activity in EPD rats. (**a**) MDA levels in gingival tissue homogenates were determined using the thiobarbituric acid-reactive substances (TBARS) assay and expressed as μM/mg tissue. (**b**) iNOS activity was determined by measuring the enzymatic conversion of L-[^3^H]-arginine to L-[^3^H]-citrulline using a radiometric assay. Data are presented as mean ± SD (*n* = 10/group). Significant vs. intact vehicle control, ** *p* < 0.01; vs. EPD control, ^#^ *p* < 0.05, ^##^ *p* < 0.01; vs. Lh-M, ^a^ *p* < 0.05, ^aa^ *p* < 0.01; vs. Lr-M, ^bb^ *p* < 0.01; vs. Sb-M, ^cc^ *p* < 0.01; vs. Bl-M, ^dd^ *p* < 0.01.

MDA levels, an indicator of lipid peroxidation, were significantly increased in the gingival tissue of the EPD control group compared with the intact vehicle control group (Figure 6a). Oral administration of Osb at all tested doses (1.25 × 10^8^, 1.25 × 10^9^, and 6.25 × 10^9^ CFU/head) significantly reduced MDA levels. Similarly, iNOS activity, a marker of nitrosative stress, was significantly elevated in EPD control rats (Figure 6b). Osb administration markedly decreased iNOS activity in a dose-dependent manner. In both MDA levels and iNOS activity, the lowest dose of Osb (Osb-L, 1.25 × 10^8^ CFU/head) showed a significantly greater reduction than the single-formula treatments (Lh, Lr, Sb, or Bl).

### 3.7. Osb Ameliorated Alveolar Bone Loss in EPD Rats

**Figure 7:**
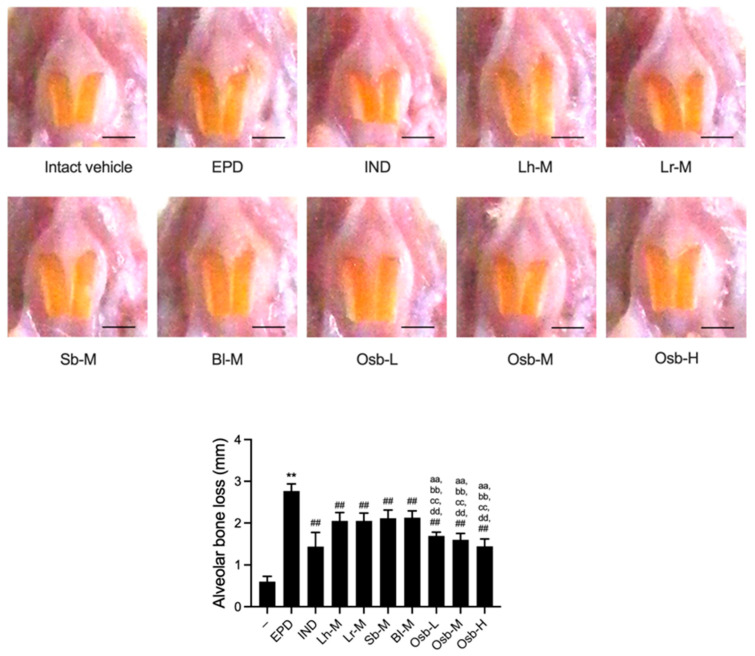
Effects of Osb on alveolar bone loss in EPD rats. Alveolar bone loss was assessed by measuring the exposed root area around the maxillary incisor region. Representative images are shown (scale bars = 2.00 mm). Data are presented as mean ± SD (*n* = 10/group). Significant vs. intact vehicle control, ** *p* < 0.01; vs. EPD control, ^##^ *p* < 0.01; vs. Lh-M, ^aa^ *p* < 0.01; vs. Lr-M, ^bb^ *p* < 0.01; vs. Sb-M, ^cc^ *p* < 0.01; vs. Bl-M, ^dd^ *p* < 0.01.

The alveolar bone loss (mm), which reflects the extent of exposed tooth roots, was markedly increased in the EPD control group compared with the intact vehicle control group (Figure 7). However, all three doses of Osb (1.25 × 10^8^, 1.25 × 10^9^, and 6.25 × 10^9^ CFU/head) significantly attenuated the ligature-induced increase in alveolar bone loss. Notably, the 1.25 × 10^8^ CFU/head dose (Osb-L) exerted a stronger protective effect than the single-formula treatments of Lh, Lr, Sb, or Bl.

### 3.8. Osb Improved Alveolar Bone Density and Suppressed Receptor Activator of Nuclear Factor-κB Ligand (RANKL)-Mediated Osteoclastogenic Signaling in EPD Rats

**Figure 8:**
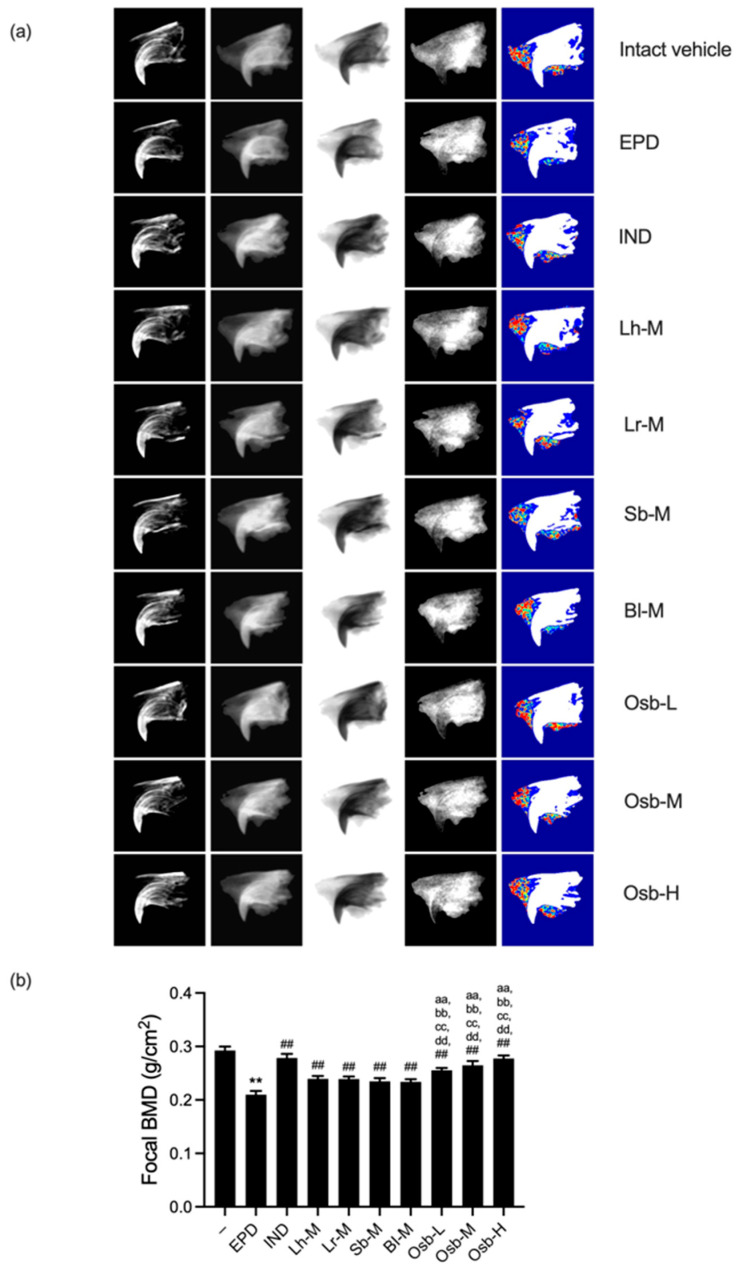
Dual-energy x-ray absorptiometry (DEXA)-based assessment of alveolar bone loss and focal bone mineral density (BMD) in EPD rats following Osb administration. (**a**) Representative DEXA images of the maxillary incisor region, captured at the end of the experimental period. (**b**) Quantification of focal BMD. Data are presented as mean ± SD (*n* = 10/group). Significant vs. intact vehicle control, ** *p* < 0.01; vs. EPD control, ^##^ *p* < 0.01; vs. Lh-M, ^aa^ *p* < 0.01; vs. Lr-M, ^bb^ *p* < 0.01; vs. Sb-M, ^cc^ *p* < 0.01; vs. Bl-M, ^dd^ *p* < 0.01.

In live DEXA analysis showed a significant reduction in focal BMD in the EPD control group compared with the intact vehicle group (Figure 8). Osb administration at all doses (1.25 × 10^8^, 1.25 × 10^9^, and 6.25 × 10^9^ CFU/head) significantly increased focal BMD relative to the EPD control group in a dose-dependent manner. Notably, the lowest dose of Osb (Osb-L, 1.25 × 10^8^ CFU/head) exerted a stronger protective effect than the single-formula treatments of Lh, Lr, Sb, or Bl.

**Figure 9:**
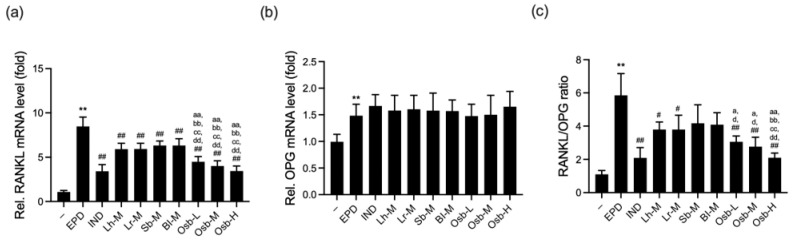
Effects of Osb on the gingival mRNA expression of receptor activator of nuclear factor-κB ligand (RANKL) and osteoprotegerin (OPG), and RANKL/OPG ratio. (**a**,**b**) mRNA levels of receptor activator of nuclear factor-κB ligand (RANKL) and osteoprotegerin (OPG) were measured by quantitative real-time PCR and expressed relative to β-actin. (**c**) The RANKL/OPG ratio was calculated by dividing the normalized RANKL expression by that of OPG. Data are presented as mean ± SD (*n* = 10/group). Significant vs. intact vehicle control, ** *p* < 0.01; vs. EPD control, ^#^ *p* < 0.05, ^##^ *p* < 0.01; vs. Lh-M, ^a^ *p* < 0.05, ^aa^ *p* < 0.01; vs. Lr-M, ^bb^ *p* < 0.01; vs. Sb-M, ^cc^ *p* < 0.01; vs. Bl-M, ^d^ *p* < 0.05, ^dd^ *p* < 0.01.

Gingival RANKL and osteoprotegerin (OPG) mRNA expression levels, as well as the RANKL/OPG ratio, were significantly elevated in the EPD control group compared with the intact vehicle control group (Figure 9). Osb administration at all doses (1.25 × 10^8^, 1.25 × 10^9^, and 6.25 × 10^9^ CFU/head) significantly reduced RANKL expression and the RANKL/OPG ratio relative to the EPD control group. However, no statistically significant changes in gingival OPG mRNA expression were observed in any test substance-administered group, including the single-formula groups, when compared with the EPD control group. Notably, the lowest dose (Osb-L, 1.25 × 10^8^ CFU/head) produced greater suppression of RANKL expression and the RANKL/OPG ratio than the single-formula treatments of Lh, Lr, Sb, and Bl.

### 3.9. Osb Ameliorated EPD-Induced Histopathological Changes in Gingival Tissue and Alveolar Bone

**Figure 10:**
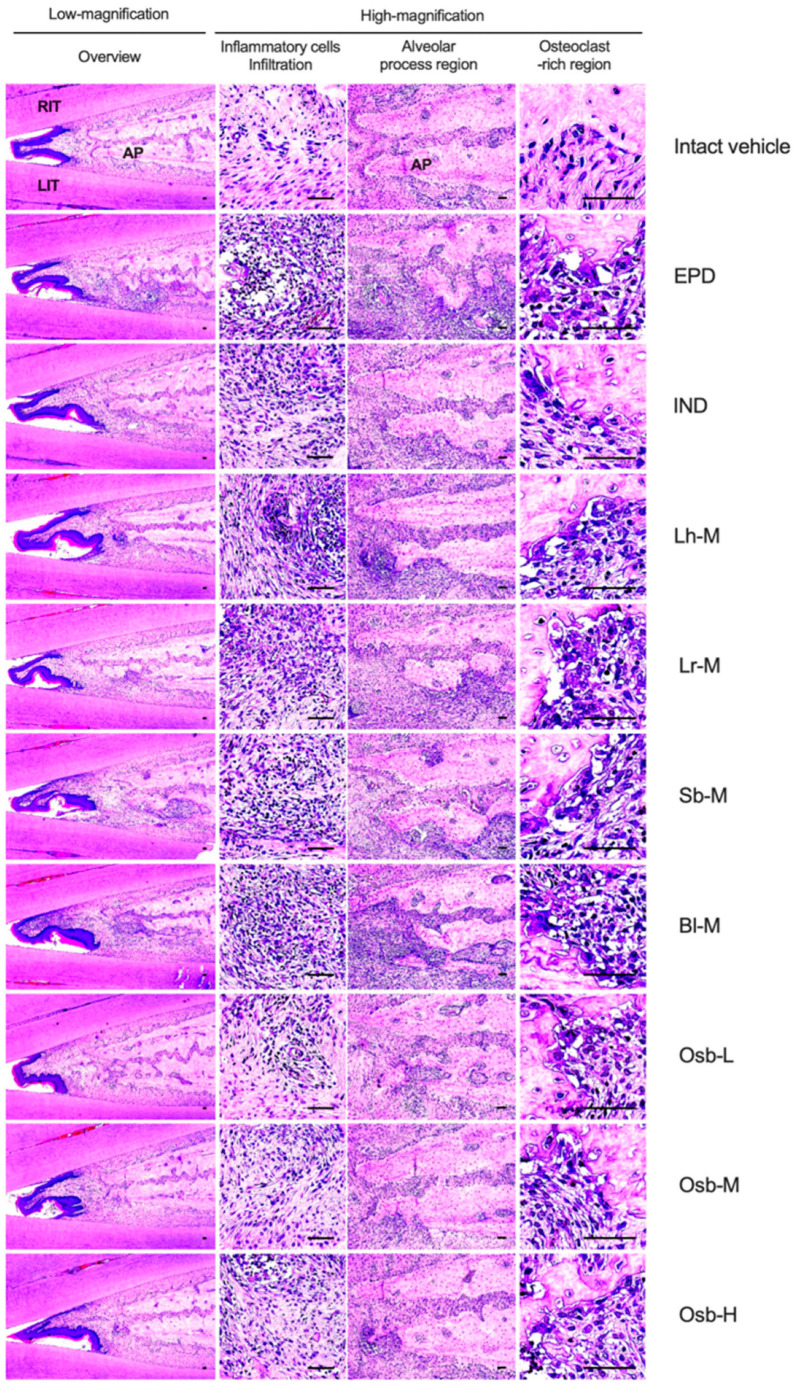
Representative histological features of the gingival tissue and alveolar bone area in EPD rats following Osb administration. Hematoxylin and eosin staining was performed on maxillary regions adjacent to the ligature site, including the gingival tissues and alveolar bone area. Images were obtained from the region between the upper incisors. Scale bars = 50 μm. RIT, Right incisor tooth; LIT, Left incisor tooth; AP, Alveolar process.

Histological evaluation was carried out in the maxillary area adjacent to the ligature site, including both the gingival tissue and the alveolar bone (Figure 10). In the EPD control group, gingival tissue showed marked signs of periodontal injury, including increased inflammatory cell infiltration, edema, and disrupted collagen organization. The structural abnormalities were reflected in substantially elevated histological scores, which assess inflammatory cell infiltration and the integrity of cementum (Table 2). Oral administration of Osb significantly improved these gingival alterations at all three doses (1.25 × 10^8^, 1.25 × 10^9^, and 6.25 × 10^9^ CFU/head). Histological scores, inflammatory cell infiltration, and collagen fiber-occupied area were significantly restored. Histopathological examination also revealed that ligature placement induced deterioration of the alveolar bone, characterized by reduced bone volume, increased osteoclast numbers, and a higher OC/BS (Table 3). Osb treatment significantly ameliorated these bone-related abnormalities in a dose-dependent manner. All three doses increased alveolar bone volume and reduced both osteoclast numbers and OC/BS. Notably, the lowest dose (Osb-L, 1.25 × 10^8^ CFU/head) produced improvements that were significantly greater than those observed with the single-formula treatments (Lh, Lr, Sb, and Bl). These results collectively indicate that Osb mitigates ligature-induced periodontal and alveolar bone damage by reducing inflammatory cell infiltration and suppressing osteoclast activation.

## 4. Discussion

The present study demonstrated that oral administration of Osb, a multi-strain probiotic formulation, exerts broad protective effects against ligature-induced EPD in rats. Periodontitis is a multifactorial disease driven by complex interactions among dysbiotic microbiota, host immune responses, and bone remodeling processes, whereas conventional therapies have largely focused on reducing either microbial burden or inflammation. In contrast, Osb treatment attenuated multiple key pathological features of periodontitis, including anaerobic bacterial overgrowth, excessive inflammatory responses, oxidative stress, epithelial barrier disruption, and alveolar bone loss. While previous clinical studies reported beneficial effects of Osb on periodontal parameters [16,17], the present study extends these findings by providing mechanistic evidence from a controlled EPD model. Notably, the combined multi-strain formulation consistently showed greater protective efficacy than individual strains administered alone, supporting that cooperative interactions among probiotic strains contribute to the enhanced therapeutic efficacy observed.

Dysbiotic shifts toward pathogenic anaerobic bacteria are recognized as an initiating factor in periodontitis, triggering excessive host inflammatory responses that drive periodontal tissue destruction [1]. Several mechanisms have been proposed for probiotic-mediated modulation of the periodontal microbiota, including competitive exclusion, competition for essential nutrients, interference with pathogen adhesion, and the production of antimicrobial substances [12]. Randomized controlled trials in patients with periodontal disease have shown that probiotics reduce the levels of key pathogens, such as *P. gingivalis*, compared with a placebo [23]. In the present study, ligature placement markedly increased anaerobic bacterial counts in buccal gingival tissue adjacent to the ligated upper incisor, whereas this increase was significantly attenuated by oral administration of Osb (Figure 2a). These findings suggest that Osb may reduce the anaerobic bacterial burden associated with ligature-induced periodontal lesions. However, the present microbiological analysis was based on cultivable anaerobic bacterial counts after 48 h of culture and was intended to compare the relative anaerobic bacterial burden among groups, rather than to comprehensively characterize the whole oral microbiota. Therefore, further studies using subgingival samples and microbiome analyses are needed to clarify changes in subgingival microbial community composition following Osb administration.

IgA serves as a first-line mucosal defense by preventing microbial adhesion, promoting bacterial agglutination, and maintaining microbial homeostasis without triggering excessive inflammation [24]. Clinical studies have shown that salivary secretory IgA levels are significantly elevated in patients with periodontitis compared with healthy individuals, indicating enhanced oral immune activation [25,26]. Consistent with these observations, gingival IgA levels were elevated in EPD control rats in the present study. Notably, Osb administration significantly reduced gingival IgA levels (Figure 2b). This decrease may be attributed to reduced antigenic stimulation resulting from the lowered burden of periodontal pathogens following probiotic treatment.

Neutrophils play a central role in host defense responses during periodontal inflammation and constitute the predominant leukocyte population infiltrating inflamed gingival tissue [27]. In periodontitis, excessive neutrophil recruitment reflects an exaggerated microbial challenge and contributes not only to bacterial clearance but also to collateral tissue damage through the release of inflammatory mediators, ROS, and MMPs [27,28,29]. Increased neutrophil proportions and neutrophil-derived markers in gingival crevicular fluid and periodontal tissues have been shown to correlate with oral inflammatory burden and the severity of periodontal disease, supporting their use as indicators of periodontal disease activity [30,31]. In the present study, Osb administration was associated with a marked reduction in neutrophil infiltration, as indicated by decreased MPO activity and lower levels of inflammatory mediators, suggesting that Osb effectively attenuates neutrophil-driven tissue responses in periodontal lesions (Figure 2c).

Sustained neutrophil activation is closely linked to extracellular matrix degradation through the release of MMPs [32]. Among these, MMP-8, a neutrophil-derived collagenase, is recognized as a key enzyme responsible for the breakdown of type I collagen and is strongly implicated in periodontal connective tissue destruction [29,31]. Consistent with this mechanism, elevated MMP-8 expression was observed in EPD control rats, suggesting enhanced extracellular matrix degradation. Osb treatment significantly reduced MMP-8 expression (Figure 4), indicating that Osb may attenuate MMP-8-associated connective tissue destruction.

The gingival epithelial barrier, also called the junctional epithelium, restricts microbial penetration and helps prevent excessive immune activation. It is stabilized by tight junction complexes composed of claudins, occludin, and scaffold proteins such as ZO-1, which seal the paracellular space and regulate the passage of ions and small solutes while limiting macromolecules and microorganisms [5]. In periodontitis, inflammatory mediators and dysbiotic bacteria such as *P. gingivalis* downregulate or proteolytically degrade junctional proteins, including claudins, occludin, ZO-1, and E-cadherin, thereby increasing epithelial permeability and enabling microbial products to infiltrate the underlying connective tissue and drive tissue destruction [7,8,33]. In the present study, ligature placement decreased the gingival mRNA expressions of tight junction-related genes, including claudin-1, claudin-5, occludin, and ZO-1, indicating compromised epithelial barrier integrity (Figure 5). Osb treatment significantly restored the mRNA expressions of these tight junction components. Preservation of the structural integrity of the gingival epithelial barrier through Osb-mediated protection of tight junction components is in line with previous probiotic findings and suggests that it may help inhibit the progression of periodontal disease by reducing the penetration of microbial products into gingival tissues.

Oxidative stress and inflammatory mediators act as major drivers of periodontal tissue destruction. Under physiological conditions, ROS generated by neutrophils and macrophages contribute to antimicrobial defense, but excessive ROS induce oxidative damage to lipids, proteins, and DNA, which amplifies inflammation, promotes MMP release, and enhances osteoclastogenesis, thereby accelerating periodontal breakdown [34]. Clinically, elevated lipid peroxidation products such as MDA in gingival crevicular fluid or saliva are positively associated with periodontitis severity, supporting a pathogenic role for oxidative stress in human disease [35,36]. In parallel, bacterial products from subgingival biofilms stimulate the production of key pro-inflammatory mediators, including TNF-α, IL-1β, and PGE_2_, which are consistently upregulated in human periodontitis and experimental periodontitis models [37,38,39]. These cytokines not only contribute directly to gingival tissue injury but also promote collagen degradation and bone loss; for example, IL-1β enhances collagenolytic MMP expression and RANKL upregulation, while TNF-α and PGE_2_ facilitate osteoclast recruitment, activation, and alveolar bone resorption [37,39,40]. In the present study, ligature-induced EPD rats showed increased gingival levels of TNF-α, IL-1β, and PGE_2_, together with elevated MDA (as a lipid peroxidation marker) and iNOS activity (as a nitrosative stress marker), consistent with exaggerated inflammatory and oxidative stress (Figure 3 and Figure 6). Osb administration significantly suppressed these inflammatory mediators and reduced MDA and iNOS activity, indicating attenuation of inflammation and oxidative damage. Taken together, these findings suggest that Osb may protect periodontal tissues by attenuating the oxidative stress and pro-inflammatory signaling that underlie progressive periodontal destruction.

Alveolar bone loss is the most serious consequence of periodontitis and the main cause of tooth loss, largely driven by osteoclast activation. This process is tightly regulated by the balance between RANKL, which promotes osteoclast differentiation, and OPG, which acts as a decoy receptor to inhibit RANKL signaling. Therefore, an increased RANKL/OPG ratio is closely associated with enhanced bone resorption in periodontitis [41]. In the present study, ligature-induced EPD led to marked alveolar bone loss (Figure 7), decreased focal BMD (Figure 8), increased osteoclast numbers and osteoclast-occupied bone surface (Table 3), and an elevated RANKL/OPG ratio (Figure 9). Osb administration effectively ameliorated these bone-related alterations, restoring focal BMD, reducing osteoclast parameters, and normalizing the RANKL mRNA level, consistent with an anti-resorptive effect on alveolar bone. Notably, the lowest Osb dose consistently produced greater bone-protective effects than each individual strain given alone at a higher dose, suggesting synergistic interactions among the combined probiotic strains. These findings support the concept that multi-strain probiotic formulations can more effectively modulate periodontal bone remodeling than single-strain approaches.

Several limitations of the present study should be considered. First, although IND was included as a pharmacological reference control for inflammation-related responses, it does not directly affect periodontal bacteria or the oral microbiota. Therefore, IND should be interpreted as an anti-inflammatory reference drug, rather than as a comparator for the microbiota-related effects of Osb. Second, an upper incisor ligature model was used in the present study, instead of the more commonly standardized molar ligature model. This incisor model was selected because it is technically simple, allows easy observation of periodontal changes, has been used in our previous studies, and is suitable as a relatively mild model for evaluating a non-drug intervention, such as a probiotic formulation [18,19,42]. However, because molar ligature models are more widely standardized, this incisor model-related limitation should be considered when interpreting the periodontal tissue and alveolar bone changes observed in the present study.

## Figures and Tables

**Table 1:** Information on primer sequences used in RT-qPCR.

Target	Orientation	Sequences (5′ → 3′)	NCBI Accession No.
RANKL	Sense Antisense	CTGATGAAAGGAGGGAGCAC, GAAGGGTTGGACACCTGAATGC	NM_057149.2
OPG	Sense Antisense	TCCTGGCACCTACCTAAAACAGCA, ACACTGGGCTGCAATACACA	U94330.1
Claudin-1	Sense Antisense	GGGACAACATCGTGACTGCT, CCACTAATGTCGCCAGACCTG	AF195500.1
Claudin-5	Sense Antisense	AAATTCTGGGTCTGGTGCTG, GCCGGTCAAGGTAACAAAGA	NM_031701.2
Occludin	Sense Antisense	CAACGGCAAAGTGAATGGCAAGAG, TCATCCACGGACAAGGTCAGAGG	NM_031329.3
ZO-1	Sense Antisense	CACGATGCTCAGAGACGAAGG, TTCTACATATGGAAGTTGGGGATC	NM_001106266.1
β-actin	Sense Antisense	TCAGGTCATCACTATCGCCAAT, AAAGAAAGGGTGTAAAACGCA	NM_031144.3

RANKL, Receptor activator of nuclear factor-κB ligand; OPG, osteoprotegerin; ZO-1, Zonula Occludens-1.

**Table 2:** Histological scoring and histomorphometric analysis of gingival tissues in the maxillary region around the ligature placement site.

Group	Histological Scores (Max = 3)	Inflammatory Cells (cells/mm^2^)
Intact vehicle	0.40 ± 0.52	32.80 ± 15.53
EPD	2.90 ± 0.32 **	644.20 ± 132.92 **
IND	1.10 ± 0.74 ^##^	130.60 ± 49.34 ^##^
Lh-M	2.10 ± 0.32 ^##^	308.40 ± 53.28 ^##^
Lr-M	2.20 ± 0.42 ^#^	313.70 ± 65.83 ^##^
Sb-M	2.30 ± 0.48	338.90 ± 62.13 ^##^
Bl-M	2.40 ± 0.52	343.40 ± 72.14 ^##^
Osb-L	1.60 ± 0.52 ^##^	198.10 ± 47.76 ^##, aa, bb, cc, dd^
Osb-M	1.40 ± 0.70 ^##^	160.50 ± 51.61 ^##, aa, bb, cc, dd^
Osb-H	1.20 ± 0.79 ^##, d^	134.60 ± 43.56 ^##, aa, bb, cc, dd^

Data are presented as mean ± SD (*n* = 10/group). Significant vs. intact vehicle control, ** *p* < 0.01; vs. EPD control, ^#^ *p* < 0.05, ^##^ *p* < 0.01; vs. Lh-M, ^aa^ *p* < 0.01; vs. Lr-M, ^bb^ *p* < 0.01; vs. Sb-M, ^cc^ *p* < 0.01; vs. Bl-M, ^d^ *p* < 0.05, ^dd^ *p* < 0.01.

**Table 3:** Histomorphometric analysis of alveolar bone area in the maxillary region around the ligature placement site.

Group	Alveolar Bone Volume (%)	Osteoclast (cells/mm^2^)	OC/BS (%)
Intact vehicle	81.19 ± 10.58	4.40 ± 2.46	2.97 ± 1.83
EPD	18.29 ± 4.99 **	60.00 ± 10.71 **	71.08 ± 12.19 **
IND	69.68 ± 8.62 ^##^	19.40 ± 6.67 ^##^	16.18 ± 6.45 ^##^
Lh-M	44.94 ± 8.45 ^##^	35.20 ± 4.24 ^##^	39.90 ± 4.56 ^##^
Lr-M	44.76 ± 7.49 ^##^	35.00 ± 3.92 ^##^	40.00 ± 3.61 ^##^
Sb-M	40.66 ± 5.99 ^##^	37.80 ± 4.37 ^##^	42.76 ± 4.95 ^##^
Bl-M	39.08 ± 7.84 ^##^	38.00 ± 4.42 ^##^	42.84 ± 4.69 ^##^
Osb-L	58.88 ± 3.06 ^##, a, bb, cc, dd^	25.60 ± 3.24 ^##, aa, bb, cc, dd^	26.97 ± 3.28 ^##, aa, bb, cc, dd^
Osb-M	64.98 ± 8.86 ^##, aa, bb, cc, dd^	22.40 ± 5.48 ^##, aa, bb, cc, dd^	22.51 ± 5.22 ^##, aa, bb, cc, dd^
Osb-H	69.54 ± 8.61 ^##, aa, bb, cc, dd^	19.80 ± 6.14 ^##, aa, bb, cc, dd^	16.45 ± 5.26 ^##, aa, bb, cc, dd^

Data are presented as mean ± SD (*n* = 10/group). Significant vs. intact vehicle control, ** *p* < 0.01; vs. EPD control, ^##^ *p* < 0.01; vs. Lh-M, ^a^ *p* < 0.05, ^aa^ *p* < 0.01; vs. Lr-M, ^bb^ *p* < 0.01; vs. Sb-M, ^cc^ *p* < 0.01; vs. Bl-M, ^dd^ *p* < 0.01.

## Data Availability

All data generated or analyzed during this study are included in this article.
